# Effect of tooth whitening strips on fatigue resistance and flexural strength of bovine dentin *in vitro*

**DOI:** 10.1371/journal.pone.0173480

**Published:** 2017-03-09

**Authors:** Laura E. Tam, Namhee Kim, Grace M. De Souza

**Affiliations:** Restorative Dentistry, Department of Clinical Sciences, Faculty of Dentistry, University of Toronto, Toronto, Ontario, Canada; The Ohio State University, UNITED STATES

## Abstract

**Objective:**

To determine the effects of whitening strips on bovine dentin fatigue resistance and flexural strength *in vitro*.

**Materials and methods:**

A total of eighty bovine dentin specimens (2x2x17mm) were treated with either: control glycerine gel on plastic film wrap or whitening strips containing 9.5% hydrogen peroxide. Treatment was applied for 30 minutes, twice a day, for 1- or 4-weeks. After the last treatment, ten specimens per group were randomly selected to undergo fatigue testing (10^6^ cycles, 3Hz, 20N) while the other ten were subjected to flexural strength testing after ten days of storage in artificial saliva. Kaplan-Meier method with a log rank test, Wilcoxon test and Cox regression were used to assess fatigue test results (p<0.05). One-way ANOVA and Tukey’s tests were used to compare the flexural strength results (p<0.05).

**Results:**

There were significant differences in survival during the fatigue test among the groups (p<0.001). Treatment (control or bleach) was a significant factor for specimen survival (p<0.001, Exp(B) = 33.45). There were significant differences in mean flexural strength (p<0.001). No significant difference was found between “1-wk control” and “4-wk control”. The mean flexural strength and fatigue resistance of the “4-wk bleach” were significantly lower than all the other groups.

**Conclusions:**

The use of whitening strips reduced the fatigue resistance and flexural strength of bovine dentin *in vitro*. Until the effect of whitening strips on mechanical properties of human dentin is fully elucidated, it remains prudent to advise patients to avoid excessive direct use of whitening strips on dentin.

## Introduction

Approximately 34% of American adults report dissatisfaction with their current tooth color [1]. Vital tooth bleaching is considered to be a conservative esthetic treatment modality to improve tooth color because no sound tooth structure is removed. Although adverse effects to enamel surface properties such as reduction in hardness [2], change in surface morphology [3] and increased susceptibility to abrasion/erosion [4] have been reported, the wide variety of different bleach concentrations and application methods, as well as different study designs, have contributed to equivocal conclusions. In general, the effects of tooth bleaching on the enamel surface have been considered to be minor or clinically insignificant [5, 6].

Less attention has been paid to the effects of bleach on dentin, with the assertion that bleach treatment is generally applied to the enamel surface and not to the dentin surface. However, it has been reported that bleach penetrates enamel and dentin readily [5, 6]. This penetration through enamel and dentin is evidenced by hydrogen peroxide measurement within the tooth pulp [7, 8] and the clinical side effect of tooth sensitivity. Furthermore, bleach can contact dentin directly in cases of dentin exposure. Gingival recession and tooth wear are common conditions which can lead to dentin exposure. The prevalence of gingival recession among adults at one or more sites ranged from 22%-100% [9–11]. 22.5% of Americans had one or more tooth surfaces with > 3mm gingival recession [9]. Tooth wear is considered a problem in both children and adults [12]. Non carious cervical lesions were identified in 17% of the teeth examined in adults [13]. The prevalence of severe wear due to abrasion, erosion and attrition was 3% at the age of 20 years and 17% at the age of 70 years [14].

Dentin constitutes the bulk of the tooth structure and acts as structural foundation for the enamel. It is therefore important to determine the effects of bleach on the mechanical properties of dentin. Alterations in the mechanical properties of dentin have been reported with both external bleach and intracoronal bleach applications. These include a significant reduction in the ultimate tensile strength [15, 16], flexural strength [17], fracture toughness [18] and stiffness [19, 20] of dentin. These *in vitro* studies suggest a possible weakening of dentin as a result of bleach treatment, at least in the short-term. Whether or not such weakening significantly increases the risk of clinical tooth fracture in the long-term is unknown.

Tooth bleaching treatment was originally only administered under the supervision of a dentist, and the bleach concentration and application was controlled. In cases of dentin exposure, dentists could minimize direct bleach application to the dentin surface by altering the tray design or by protecting exposed dentin surfaces with a restorative material. Many of the bleach studies that supported the safety and effectiveness of the vital tooth bleaching were conducted using concentrations of hydrogen peroxide equivalent to approximately 3.5% using a tray-delivery method for a limited period of time [21, 22]. Less is known about the effects of bleach used in higher concentrations, in prolonged applications, in multiple applications that simulates periodic bleach re-treatment and in over-the-counter delivery systems, and the research on the short- and long-term safety of tooth whitening products and procedures remains as one of the American Dental Associations research objectives [23].

Currently, a great variety of tooth bleaching products are available over-the-counter. A popular over-the-counter product utilizes strips to deliver hydrogen peroxide to the tooth surface. It generated $166 million in retail sales in 2002 and has maintained a healthy growth rate [24], which suggests that the annual number of new or repeat users of this product ranges in the millions. The strips are a proprietary polymer-based adhesive matrix with claimed optimized adhesive and cohesive properties. The hydrogen peroxide concentrations on whitening strips are generally higher than 3.5% but the volumes used are significantly smaller than what is used in bleaching trays. The smaller volume is extremely advantageous with regard to minimizing peak salivary hydrogen peroxide concentration and toxicity. A review paper evaluated the effectiveness and side effects of over-the-counter and dentist-dispensed chemically-based tooth whitening products designed for home use [25]. It reported differences in efficacy between the tooth whitening products designed for home use, mainly due to the levels of active ingredients, and that all trials were short and at high risk of bias. Whitening strips were considered to be effective [25], although perhaps less efficient than at-home tray-bleaching [26, 27]. Whitening strips and products with high hydrogen peroxide concentration caused more users to complain of tooth sensitivity [25].

It is possible that the clinical effects of excessive tooth bleaching on dentin and the tooth structure are not immediately apparent and that incidences of tooth fractures attributable to tooth bleaching will appear much later in susceptible teeth after years of function. Flaws in dentin on a microscopic level, potentially caused by bleach treatment, may propagate over time. Fatigue tests are more useful than strength tests to assess structural longevity [28]. A recent *in vitro* study reported a significant decrease in the fatigue resistance of bovine dentin following the use of a tray-delivered type of bleach application (10% carbamide peroxide, six hours a day for 2- or 8-weeks) [29]. In comparison to a tray-delivered type of bleach application, whitening strips generally utilize a hydrogen peroxide formulation rather than a carbamide peroxide formulation, a higher concentration of bleach, a smaller volume of bleach, a shorter bleach application time, and a thin polymer strip rather than a full contour tray. The effect of whitening strips on the fatigue resistance and flexural strength of dentin is unknown and cannot be extrapolated from tray-bleaching studies.

This study provides new information related to the long-term effects of multiple applications of whitening strips on tooth structural integrity. The objective of this study was to determine the effects of whitening strips on the fatigue resistance and flexural strength of bovine dentin *in vitro*. The null hypotheses for this study were: 1) strip bleaching has no effect on the fatigue resistance of bovine dentin *in vitro*, and 2) strip bleaching has no effect on the fatigue resistance of bovine dentin *in vitro*.

## Materials and methods

Bovine incisors (extracted within twelve months of the experiment, frozen until use) were used to provide the dentin for testing. Ethical review was not required for the use of bovine teeth taken from animals slaughtered for human food consumption (Research Oversight and Compliance Office, University of Toronto). One dentin specimen was obtained from each tooth. Rectangular beams, approximately 2x2x17mm, were prepared from the dentin on the facial surface using a water-cooled low-speed diamond saw (Buehler Limited, Lake Bluff, IL, USA), keeping the location and orientation of the dentin as standardized as possible. A micrometer (Digimatic Caliper, Mitutoyo Corporation, Kanagawa, Japan) was used to measure specimen dimensions to the nearest 0.01mm. The dentin specimens were stored in freshly-made artificial saliva, pH 7.2, which was changed daily, in individual containers until testing. The composition of the artificial saliva is listed in Table 1 [30].

**Table 1:** Composition of artificial saliva [30].

Chemical component	Concentration
Methyl-p-hydroxybenzoate	2.00 g/l
Na carboxymethylcellulose	10.0 g/l
MgCl_2_^.^ 6 H_2_0	0.29 mM
CaCl_2_^.^ 2 H_2_0	1.13 mM
K2HPO4	2.40 mM
KCl	8.38 mM
F	0.05 ppm

The specimens were randomly divided into two groups of 40 each: control treatment or bleach treatment as outlined in Table 2. In the control treatment group, a glycerine gel (Placebo, Ultradent Products, South Jordan, UT, USA) was syringed onto plastic film wrap (Cling Wrap, The Glad Products Company, Oakland, CA, USA) to form an approximately 1-mm thick layer. The plastic film was then wrapped around the dentin specimen and visual confirmation of contact between the treatment gel and the entire surface dentin was made. In the bleach treatment group, a whitening strip containing 9.5% hydrogen peroxide (Crest 3D White Whitestrips Advanced Vivid, Proctor & Gamble Incorporated, Mason, OH, USA) was cut to size and wrapped around the dentin specimen. Both groups were exposed to the treatment for 30 minutes twice a day for 1- or 4-weeks. The specimens were stored in an incubator (Model 630D, Fisher Scientific Company, Hampton, NH, USA) at 37°C, >80% relative humidity for the duration of the treatment. At the end of each treatment, the specimens were rinsed with tap water to remove all external traces of treatment gel and were stored in 37°C artificial saliva until the next treatment.

**Table 2:** Outline of test groups.

Group	Treatment	Time
1-wk control	Control glycerine gel in plastic film	30 minutes twice daily for 7 days
4-wk control	Control glycerine gel in plastic film	30 minutes twice daily for 28 days
1-wk bleach	Whitening strip containing 9.5% hydrogen peroxide	30 minutes twice daily for 7 days
4-wk bleach	Whitening strip containing 9.5% hydrogen peroxide	30 minutes twice daily for 28 days

“wk” = week.

At the end of the last treatment, ten specimens from each group were randomly selected for fatigue testing. These ten dentin specimens were mounted in custom-designed holders with a span distance of 8mm, immersed into 37°C artificial saliva, and subjected to fatigue loading at 20N and 3Hz for up to 10^6^ cycles using a chewing simulator (CS 4.4 Chewing Simulator, SD Mechatronik, Feldkirchen-Westerham, Germany). The completion of the 10^6^ cycles took approximately 3.8 days for each specimen unless the specimen fractured prematurely, at which time the cycle at failure was recorded.

The ten specimens that were not selected for fatigue testing were stored in 37°C artificial saliva, which was changed daily, for ten days. These specimens were mounted on an Instron universal testing machine (Model 4301, Instron Corporation, Canton, MA, USA) for three-point bending flexural strength test using a custom-designed mounting jig with a span distance of 14mm. During testing, the mounting apparatus was immersed in a 37±2°C custom-built water bath. Flexural loading was applied at a rate of 0.5 mm/minute until specimen fracture. The force recorded at fracture was used to calculate flexural strength.

IBM SPSS Software 24 (International Business Machines Corporation, Armonk, NY, USA) was used for statistical analyses. One-way ANOVA and Tukey’s tests were used to compare the flexural strength results (p<0.05). For the fatigue test results, the Kaplan-Meier method with a log rank test was used to determine whether the time to failure differed (p<0.05). The Wilcoxon test was used for pairwise comparison and Cox regression was used to assess the relationship between survival time and the covariates (treatment and time) (p<0.05).

## Results

One specimen broke accidentally from the “4-wk bleach” flexural strength test group during handling, leaving 39 specimens for flexural strength testing and 40 specimens for fatigue testing.

### Fatigue resistance

**Fig 1:**
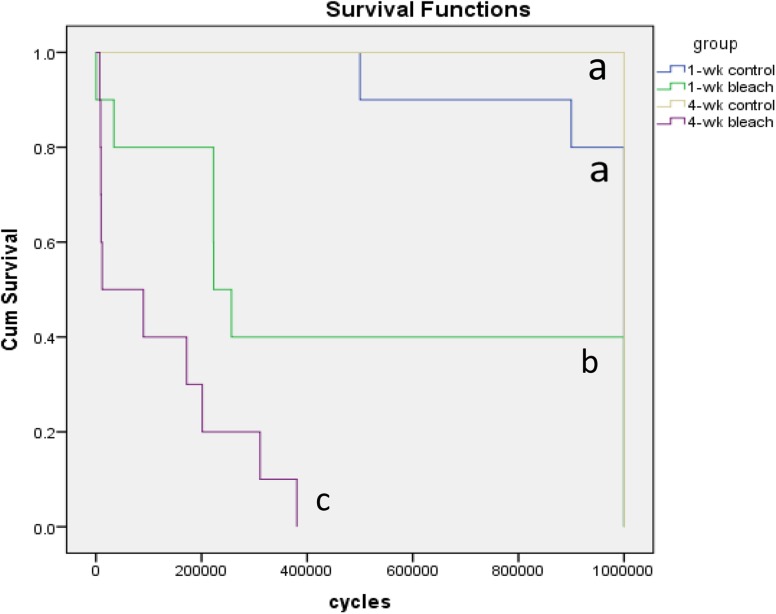
Survival plot. The cumulative survival of the specimens over the number of cycles during the fatigue test is shown. The survival distributions for the interventions was significantly different (p<0.001). Groups denoted by the same letter indicates no significant difference (p<0.05).

The median number of cycles survived by the “1-wk bleach” and “4-wk bleach” specimens was 0.22 X 10^6^ and 0.01 X 10^6^ respectively, while the median number of cycles survived by the specimens in both control groups was 1.00 X 10^6^. Fig 1 shows the cumulative survival of the specimens plotted against the number of cycles during the fatigue test in a survival functions plot. The survival distributions for the interventions was significantly different (p<0.001). The “4-wk bleach” group had a 100% premature failure rate and the lowest survival distribution. There was no significant difference in the survival distributions between the control groups, which were both significantly higher than the bleach groups. Cox regression suggested that time (1-week or 4-weeks of treatment) was not a significant factor for specimen survival (p = 0.065, Exp(B) = 2.51). Treatment (control treatment or bleach treatment) was a significant factor for specimen survival (p<0.001, Exp(B) = 33.45).

### Flexural strength

**Fig 2:**
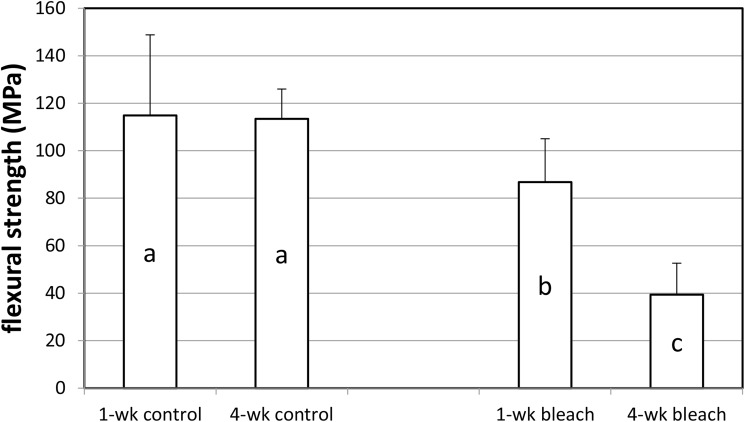
Mean flexural strength results (± standard deviation). There were significant differences in mean flexural strength among the groups (p<0.001). Groups denoted by the same letter indicates no significant difference (p<0.05).

The mean flexural strength results are shown in Fig 2. There were significant differences in flexural strength values (p<0.001). No significant difference was found between “1-wk control” and “4-wk control”. The mean flexural strength of the “4-wk bleach” was significantly lower than the mean flexural strength of all the other groups.

## Discussion

Based upon the results of this study, the null hypotheses, 1) strip bleaching has no effect on the fatigue resistance of bovine dentin *in vitro*, and 2) strip bleaching has no effect on the fatigue resistance of bovine dentin *in vitro*, were rejected.

Bovine incisors were used as the source of dentin primarily because human teeth are too small to provide the size of dentin needed for the flexural strength and fatigue tests. Although human dentin is a more clinically relevant substrate, human teeth are more difficult to obtain, may have larger variations with respect to age, diet and environment, are too small to produce larger specimen sizes, and may be associated with a greater ethical burden. Therefore, bovine dentin is widely used as a substitute for human dentin. Recent papers reviewed the use of bovine teeth as a substitute for human teeth in dental research and confirmed that bovine dentin is widely used in dental research. Although bovine teeth can be a reliable substitute for bond strength studies to dentin [31], there were inconsistencies in the literature comparing human and bovine teeth and it was concluded that differences in morphology, chemical composition and physical properties between human and bovine dentin must be considered when interpreting results obtained when bovine teeth are used [32]. With regard to mechanical properties, no significant differences were reported between human and bovine dentin for fatigue, ultimate tensile strength and modulus of elasticity [33, 34]. Bovine dentin has used in other papers studying the effect of bleach on dentin mechanical properties [17, 19, 35]. Previous studies have shown that frozen storage did not significantly affect the tensile strength of or the shear bond strengths to bovine dentin [34, 36]. The reported effect of bleach on bovine dentin structural integrity (flexural strength) has been shown to be similar to the reported effect of bleach on human dentin structural integrity (fracture toughness) [18, 37]. Therefore, it is expected that the decrease in flexural strength and fatigue resistance that was observed in this study on bleached bovine dentin would be comparable on human dentin.

The use of whitening strips was associated with a significant reduction in fatigue resistance in this *in vitro* study. Significantly fewer bleached specimens than control specimens survived the 10^6^ cycles and they failed significantly earlier. The majority of the control group specimens survived the entire fatigue test. This was expected as the fatigue test was considered to be equivalent to approximately one or two years of masticatory loading cycles by a normal adult and below the endurance limit of dentin [28, 38]. In contrast, only four (40%) “1-wk bleach” specimen and none of the “4-wk bleach” specimens survived the entire fatigue test. The most fatigue-resistant specimen of the “4-wk bleach” group managed to survive only 0.38 X 10^6^cycles whereas the least fatigue-resistant specimen of the “1-wk control” group survived 0.50 X 10^6^ cycles. The value of the hazard ratio (Exp(B) = 33.45) suggested that the hazard rate in the bleach treatment groups was 33 times higher than in the control groups.

The use of the whitening strips was also associated with a reduction in flexural strength in this *in vitro* study. After 1 week of twice daily whitening strip applications, the mean flexural strength of bovine dentin was 20% lower than the 1-wk control. After 4 weeks of twice daily whitening strip applications, the mean flexural strength of dentin was significantly lower than the 4-wk control, by more than 60%. It has been shown that there is residual hydrogen peroxide in the tooth structure after bleach treatment, enough to significantly impair bonding of composite resin to enamel that has just received bleach treatment [39]. Bond strength returns to normal values if bonding is delayed by approximately 7 days, as it has been shown that hydrogen peroxide completely leached from enamel within 7 days after bleach application [40]. In this study, the flexural strength test was performed ten days after the last bleach treatment. This was done to avoid or reduce the possibility of temporary effects such as those reported in bond strength studies from residual hydrogen peroxide within the tooth structure. The reduced flexural strength results for the bleached dentin ten days after the end of bleach treatment in this study suggested that the reduction in flexural strength was not a temporary reduction due to residual hydrogen peroxide within the dentin. The ability for recovery of flexural strength over time is unknown.

This study confirms the findings of a previous paper which reported an adverse effect on the same mechanical properties of bovine dentin after simulated night-time tray-bleaching [29]. There was a reduction in dentin flexural strength and fracture resistance when 10% carbamide peroxide was applied in a simulated tray-bleaching technique for a total time of 84 or 288 hours [29]. In comparison to night-time tray-bleaching, whitening strips are used for a much shorter duration each time. In this study, whitening strips were applied to dentin for a total time of 7 hours in the “1-wk bleach” and 28 hours in the “4-wk bleach” group. The effect of the application of the whitening strips for these shorter time periods on bovine dentin flexural strength and fatigue resistance was still highly significant. This might be explained by the higher hydrogen peroxide concentration in the whitening strip, the twice-daily application of the whitening strip, and the lower pH [41, 42] of the bleach gel in the whitening strip compared with the at-home tray bleach.

It is possible that a prolonged period of time is not required for each bleach application to cause a reduction in dentin mechanical properties if much of the effects of bleach occur within the initial period of each bleach application. In that case, the frequency or the total number of applications could be a more significant factor than the total duration of application. The recommended number of applications per day for strip whitening is generally once a day, but the product instructions suggest to use it twice a day for faster results. This study used the higher twice-daily application frequency and did not test once-daily application frequency. The total number of applications in the “1-wk bleach” group was 14 times, equivalent to the maximum use of a standard package containing 14 whitening strips for each dental arch. The total number of applications was 56 times in the “4-wk bleach” group, equivalent to a prolonged course of tooth bleaching The progressively lower fatigue resistance and flexural strength results for the “4-wk bleach” group compared to the “1-wk bleach” group and control groups suggested that the total number of bleach applications should be kept to a minimum whenever possible.

The mechanism of how teeth are whitened by hydrogen peroxide is not fully understood. It has been postulated that hydrogen peroxide oxidizes color producing materials within the tooth [43]. This suggested mechanism could leave the bleached tooth structure intact, as the target of bleach oxidation is not the tooth structure itself. A more recent proposal for the mechanism of tooth whitening was related changes to the organic component of enamel, with a resultant more opaque appearance of the tooth organic component [44]. This proposed mechanism could partly explain the reduction in dentin mechanical properties observed this study, as the target of bleach oxidation would be an integral part of the tooth structure rather than a separate color producing material. If the organic component is significantly affected by hydrogen peroxide as suggested by a few studies [19, 45, 46], hydrogen peroxide may have an even greater adverse effect on dentin mechanical properties than enamel, as dentin contains a relatively higher organic content than enamel.

In the clinical environment, the dentin would not be as affected by the bleach in the whitening strips as it was in this *in vitro* study because in the clinical environment, saliva can buffer, dilute or wash away the bleaching agent, intraoral movements can displace the whitening strips, dentin is protected by overlying enamel, the cross-section of dentin is bulkier, and dentin may have the ability to remineralize or repair. The clinical environment has been given as the primary reason why no significant difference in dentin fracture toughness was determined when bleach was applied *in situ* [47]. The clinical significance of the effects of whitening strips on the mechanical properties of dentin reported herein may be arguable with regards to ultimate tooth longevity. Other cosmetic dental procedures that require tooth structure removal, such as veneers or crowns, would have a much more significant impact on tooth structural integrity than tooth bleaching. However, the possible long-term effect of excessive bleaching on the mechanical properties of the tooth structure should not be ignored. Whitening strips can effectively and conveniently lighten teeth at a significantly lower cost than dentist-supervised bleach treatments. They can therefore be more prone to excessive or chronic use. Consumers should be educated about the possible long-term risks to tooth structure caused by excessive repeated or prolonged use of whitening strips, especially when applied directly to dentin.

## Conclusions

Although tooth bleaching products are generally considered safe and effective when used as directed, the observed reduced fatigue resistance and flexural strength of dentin exposed to simulated repeated whitening strips treatments *in vitro* raise concern for the structural integrity and longevity of dentin. Until the effect of whitening strips and extensive bleaching on the long-term mechanical properties of human dentin is fully elucidated, it remains prudent to advise patients to avoid excessive use of whitening strips directly on dentin.

## Supporting information

S1 Dataset Dataset for fatigue and flexural strength tests. (XLSX)
